# Counts, Characteristics and Outcomes of Patients Transported by the Royal Flying Doctor Service to Metropolitan Perth With Suspected Acute Coronary Syndrome: Western Australian Linked Data Study

**DOI:** 10.1111/ajr.70093

**Published:** 2025-09-23

**Authors:** Julian Ming, Helen Bartholomew, David Preen, John Fisher, Tom Briffa, Andrew Hooper, James M. Rankin, Abdul Rahman Ihdayhid, Derrick Lopez

**Affiliations:** ^1^ School of Population and Global Health The University of Western Australia Crawley Western Australia Australia; ^2^ Royal Flying Doctor Service Western Operations Jandakot Western Australia Australia; ^3^ Fiona Stanley Hospital Murdoch Western Australia Australia; ^4^ Harry Perkins Institute of Medical Research Perth Western Australia Australia; ^5^ Curtin Medical School Curtin University Perth Western Australia Australia

**Keywords:** acute coronary syndrome, aeromedical transport, diagnostic coronary angiography, interventional coronary procedure, Royal Flying Doctor Service, rural health services, survival, Western Australia

## Abstract

**Objectives:**

Determine counts, characteristics and outcomes following transport by the Royal Flying Doctor Service Western Operations (RFDSWO) to Perth.

**Study Design:**

Retrospective cohort study of the RFDSWO aeromedical patient dataset linked to administrative datasets.

**Participants:**

Suspected acute coronary syndrome (ACS) patients aged ≥ 25 years transported from rural Western Australia to Perth between 2001 and 2017.

**Main Outcome Measures:**

Basic counts and proportions. Poisson regression was used to determine absolute change in number of transports and relative risk (RR) of receiving diagnostic coronary angiography; logistic regression to model odds ratio (OR) of death between transport and end of hospital care.

**Results:**

RFDSWO carried out 11 390 transports for suspected ACS between 2001 and 2017, with the absolute number of annual transports increasing by 6.0%. After excluding 164 transports without linked records, the remaining 11 226 consisted of patients with a mean age of 60.3 years and 70.8% male. Most (99.1%) were hospitalised and 1.8% died. Among those hospitalised, 84.5% received diagnostic coronary angiography and 74.5% were discharged with a diagnosis of ACS. Females (RR = 0.97; CI = 0.95–0.99), higher comorbidity scores, and those from the Pilbara/Midwest/Wheatbelt regions (compared to South West) were less likely to receive diagnostic coronary angiography. Older patients (OR = 1.07; CI = 1.06–1.11), earlier transport years, higher comorbidity scores, those with priority 1 transport or requiring medical escort, and those from the Kimberley (compared to South West) were more likely to die.

**Conclusions:**

Findings suggest a high suspicion of coronary artery pathology among transported patients. Patient‐level and regional differences in outcomes warrant further investigation with more granular data.

**Summary**

- What this paper adds?
  ○ There was a temporal increase in the number of transfers for suspected ACS in the era of declining burden of ACS in Australia. Findings suggest a high suspicion of coronary artery pathology among transported patients.
- What is already known on this subject?
  ○ RFDSWO provides aeromedical transport to rural WA people, including those experiencing ACS; however, outcomes for these patients collectively following transport are unknown.

## 1 Introduction

Acute coronary syndrome (ACS) is one of the leading causes of morbidity and mortality in Australia [1]. People living in rural and remote Western Australia (WA) generally experience poorer health than their metropolitan counterparts. Rural people have greater morbidity and disease risk factors such as smoking, overweight and obesity; reduced access to, and utilisation of, health services; travel greater distances to medical services; and experience significantly greater delays in emergency medical care and tertiary management [2, 3, 4]. In WA, the largest and one of the most sparsely populated Australian states, the provision of aeromedical primary retrieval and interhospital transfer services by the Royal Flying Doctor Service Western Operations (RFDSWO) aims to address this health inequity by minimising delays to advanced care in tertiary hospitals in the state capital Perth [5, 6].

People experiencing suspected ACS require timely access to emergency services to receive guideline‐indicated care, including coronary reperfusion therapy at a capable hospital site. In rural and remote WA, timely invasive intervention is complicated by limited local medical services, variable road access and protracted distances to tertiary hospitals, with Perth and Bunbury hospitals being the only cardiology‐capable centres. RFDSWO services more than 2.5 million square kilometres, providing round‐the‐clock primary retrieval or inter‐hospital transfer of people from rural and remote areas requiring tertiary hospital care.

The care received and outcomes after such transport are largely unknown to RFDSWO. Therefore, in this study of patients transported by RFDSWO to Perth for a suspected ACS between 2001 and 2017, we aimed to determine: (i) the number of patients transported and the change over time; (ii) patient and transport characteristics; (iii) outcomes following transport; and (iv) principal discharge diagnosis from the Perth hospital. These findings will provide insights into the continuity of care for rural and remote Western Australians with suspected ACS flown to Perth and inform policy and practice, with the aim of improving service provision to rural and remote residents.

## 2 Methods

### 2.1 Setting

WA has a population of 2.8 million residents in 2022, representing 10% of the national population [7]. The straight‐line distance to Perth from Kununurra, the furthest major town, is 2200 km. Most (98%) RFDSWO aeromedical transports to Perth arrived at Jandakot Airport and a small number at Perth Airport, both < 30 min to tertiary hospitals by road. Rural WA health services are administered within seven geographical regions defined by the WA Health Department. Average transport times (hours) from rural health regions to Perth are: Kimberley = 6.5, Pilbara = 4.0, Midwest = 1.6, Goldfields = 2.1, Wheatbelt = 0.9, South West = 0.8 and Great Southern = 2.1.

### 2.2 Study Population

Our study population was patients aged ≥ 25 years who were transported by RFDSWO from the seven aforementioned regions to Perth for a suspected ACS (International Classification of Diseases, Ninth Revision, Clinical Modification [ICD‐9‐CM]: 410, 411.1; International Classification of Diseases, Tenth Revision, Australian Modification [ICD‐10‐AM]: I20.0, I21) between 1 January 2001 and 31 December 2017. Patients were categorised as suspected ACS by RFDS clinicians based on clinical history and findings from the time of referral to transport completion.

### 2.3 Data Sources

The study population was identified from the aeromedical patient dataset maintained by RFDSWO, which included variables like departure and arrival times, diagnosis, priority, medical escort (flight doctor in addition to flight nurse) required, originating health region and in‐flight death. We accessed the following unit‐record linked data relating to the study population: (i) Hospital Morbidity Data Collection (HMDC) covering all public and private hospitalisations in WA from 1985 to 2020; (ii) Emergency Department Data Collection (EDDC) covering all presentations in WA from 2002 to 2019; (iii) WA Death Registrations covering all deaths (only month and year of death provided) in the state from 2001 to 2019; and (iv) Electoral Roll (covering all elector registrations from January 2000 to September 2020). These datasets are maintained by the WA Department of Health and linked through the WA Data Linkage System (WADLS) [8]. Earlier research showed that the proportions of invalid (false positives) or missed links (false negatives) are both estimated at 0.1% of matches [8] and this has been confirmed in a recent report by WADLS [9].

### 2.4 Number of Patients Transported and Change Over Time

We determined the annual number of transports from the RFDSWO ‘aeromedical patient dataset’. We excluded any rural‐to‐rural transfers that occurred before a patient was transported to Perth. We used Poisson regression with population counts from the Australian Bureau of Statistics as the denominator to determine the annual increase in the number of ACS transports, where the exponentiated coefficients from the year variable represented the percentage annual increase in the number of transports.

### 2.5 Patient and Transport Characteristics

We determined patient age, sex, rural health region, transport priority, medical escort and RFDSWO diagnosis by ACS sub‐type (ST‐elevation myocardial infarction [STEMI]; non‐ST‐elevation myocardial infarction [NSTEMI]; unstable angina [UA]) (Table S1) as coded by RFDSWO. We also determined their previous medical history (with a fixed 10‐year lookback from transport date) and Charlson comorbidity score [10] from the principal and secondary discharge diagnosis fields in the HMDC.

### 2.6 Outcomes Following Transport to Perth

Using the HMDC, EDDC and Death Registrations, we determined if patients survived the RFDSWO transportation episode, defined as the period from the start of the transportation and one of the following which occurs first: (i) depart RFDSWO care (either alive or dead) without subsequent treatment in the Emergency Department (ED) or hospital ward within a day of arrival in Perth, (ii) depart ED (either alive or dead) without subsequent hospitalisation within a day of arrival in Perth, or (iii) discharge from hospital (either alive or dead) when admitted within a day of arrival in Perth (Figure S1). We also determined whether patients were treated in the metropolitan emergency department (ED) only or admitted to a hospital ward within a day of arrival in Perth. Among those who were admitted, we determined their length of stay (LOS), coronary artery procedures performed (Table S1), type of admission (acute vs. other) and survival status at discharge. Hospitalisation episode and LOS included transfers between metropolitan hospitals consistent with our earlier studies [2, 11, 12]. Rehabilitation was determined from the principal discharge diagnosis field in the HMDC (ICD‐10‐AM: Z50).

### 2.7 Principal Discharge Diagnosis From Metropolitan Hospital

Among those who were admitted to hospital following transport to Perth, we determined their principal discharge diagnosis from the HMDC. If the patient was transferred between metropolitan hospitals, we used the diagnosis from the first metropolitan hospital as this represented the acute ACS presentation and subsequent transfers largely represented rehabilitation care.

### 2.8 Statistical Analysis

Descriptive results are presented as counts and proportions for categorical variables and either mean (with standard deviation [SD] or 95% confidence interval [CI]) or median (with 25th, 75th percentiles) depending on the distribution of the data. We compared characteristics by survival status following transport using *t*‐tests for continuous and Chi‐squared analyses for categorical data. As some patients had more than one transport during the study period, we used multilevel multivariable logistic regression (which allows for clustering of events within patients as these events are not independent; e.g., patients must survive the first transport event in order to have a subsequent transport) to determine the association (odds ratio [OR]) between patient and transport characteristics and survival to hospital discharge. As the outcome of receipt of coronary artery procedure was not rare (> 10%) [13], we used multilevel multivariable Poisson regression with robust standard errors, consistent with our earlier studies [2, 14, 15], to determine the association (risk ratios [RR]) between patient and transport characteristics and whether these procedures were performed. In this model, the Poisson regression estimates RR for binary outcomes (i.e., procedure performed vs. not performed).

For sensitivity analysis, we restricted data to the last 5 years of the study (2013–2017) to determine whether the findings are generalisable over the entire study period. Data analyses were performed using Stata [16]. *p*‐values < 0.05 were considered statistically significant.

### 2.9 Ethical Consideration

Human Research Ethics Committee approval for the project was obtained from the (RGS0000003686) and (RA/4/20/6520).

## 3 Results

### 3.1 Number of Patients Transported and Change Over Time

Between 2001 and 2017, there were 11 390 transports from all WA rural health regions to Perth for a diagnosis of suspected ACS (Figure S1). We excluded 164 transports for which there were no records that linked to any of the aforementioned datasets. Plausible reasons include admission to a non‐hospital setting, non‐WA residents and patient decision against medical treatment. After excluding these patients, our final study included 11 226 transports for 9874 unique patients. There were 298 transports in 2001 and 831 in 2017 (Table 1). The number of transports increased for every region except for the South West, where numbers declined from 2013 (Figure S2). Overall, the absolute number of transports for suspected ACS increased by 6.0% [CI = 5.6%–6.5%] per year between 2001 and 2017.

**TABLE 1:** Characteristics of RFDSWO transports to Perth for suspected ACS between 2001 and 2017 (*N* = 11 226 transports representing 9874 unique patients).

Number of transports, *n* (%)	
2001	298 (2.7)
2002	326 (2.9)
2003	321 (2.9)
2004	433 (3.9)
2005	430 (3.8)
2006	473 (4.2)
2007	530 (4.7)
2008	561 (5.0)
2009	625 (5.6)
2010	710 (6.3)
2011	879 (7.8)
2012	950 (8.5)
2013	1052 (9.4)
2014	968 (8.6)
2015	984 (8.8)
2016	855 (7.6)
2017	831 (7.4)
Patient characteristics	
Age in years at transport, mean (SD)	60.3 (13.0)
Sex: male, *n* (%)	7945 (70.8)
Medical history, *n* (%)	
Myocardial infarction	1993 (11.8)
Congestive heart failure	991 (8.8)
Peripheral vascular disease	702 (8.3)
Cerebrovascular disease	562 (5.0)
Chronic pulmonary disease	41 (0.4)
Rheumatologic disease	118 (1.1)
Peptic ulcer disease	218 (1.9)
Liver disease	254 (2.3)
Diabetes	2494 (22.2)
Hemiplegia or paraplegia	218 (1.9)
Renal disease	761 (6.8)
Malignancy, including metastasis	651 (5.8)
Charlson comorbidity score (points), *n* (%)	
0	6450 (57.5)
1–2	3036 (27.0)
≥ 3	1740 (15.5)
ACS sub‐type in RFDSWO dataset	
STEMI	1849 (16.5)
NSTEMI	3385 (30.2)
Unspecified MI	2255 (20.1)
Unstable angina	3737 (33.3)
Rural health region, *n* (%)	
Kimberley	907 (8.1)
Pilbara	1326 (11.8)
Midwest	2101 (18.7)
Wheatbelt	1053 (9.4)
Goldfields	1416 (12.6)
South West	3050 (9.4)
Great Southern	1373 (12.2)
Transport characteristics	
Priority 1 (highest priority), *n* (%)	830 (7.4)
Doctor required, *n* (%)	7649 (68.1)

*Note:* Higher Charlson comorbidity score indicates higher comorbidity burden.

Abbreviations: ACS = acute coronary syndrome, MI = myocardial infarction, NSTEMI = non‐ST‐elevation myocardial infarction, RFDSWO = Royal Flying Doctor Service Western Operations, STEMI = ST‐elevation myocardial infarction.

### 3.2 Patient and Transport Characteristics

The average age of transported patients was 60.3 years (SD = 13.0) and 70.8% (*n* = 7945) were male (Table 1). Twenty‐two percent (*n* = 2494) of transports had a history of diabetes and 11.8% (*n* = 1993) prior myocardial infarction (MI). Around 19% (*n* = 2101) of transports originated from the Midwest while only 8.1% (*n* = 907) were from the Kimberley region. Over two‐thirds of transports required a medical escort (*n* = 7649) and 7.4% (*n* = 830) were classified as Priority 1 (highest priority).

Prior to 2008, RFDSWO diagnosis codes did not distinguish the different subtypes of MI. Since the inclusion of MI subtypes in 2008, the number of transported cases diagnosed with NSTEMI increased over time (Figure S3).

### 3.3 Outcomes Following Transport to Perth

Seven of the 11 226 transports (0.1%) resulted in death before reaching a Perth hospital (Figure S1). Of the remaining 11 219 transports, 32 (0.3%) were not seen in the Emergency Department (ED) or hospitalised, 61 (0.5%) were managed only in the ED and 11 126 (99.2%) were admitted to hospital. Nine of the 61 transports (14.8%) that were managed only in the ED died, whilst the remaining were discharged alive. Separately, 10 936 (98.3%) of the hospitalised patients were discharged alive. Patients who died (*n* = 206) were older (70.7 vs. 60.1 years; *p* < 0.001), more likely to require Priority 1 transport (15.5% vs. 7.2%) and required a medical escort (85.0% vs. 67.8%) than those who survived (Table S2). The multivariable logistic regression model (Table 2) shows the independent associations between age (OR = 1.07; CI = 1.06–1.11), transport year (OR = 0.48; CI = 0.31–0.75 for 2013–2017 compared to 2001–2006), Charlson comorbidity score (OR = 3.36; CI: 1.72–6.54 for scores ≥ 3 compared to 0), Priority 1 transport (OR = 2.92; CI = 1.78–4.79), medical escort required (OR = 3.22; CI = 1.88–5.51), rural health region (OR = 4.90; CI = 2.23–10.78 for Kimberley compared to the South West) and death. This model highlights older age, greater comorbidity, higher acuity transport categorisation and more remote region as predictive factors for in‐hospital mortality.

**TABLE 2:** Independent associations between patient/transport characteristics and death during the RFDSWO transportation episode for suspected ACS between 2001 and 2017 (*N* = 11 226).

	Odds ratio (95% CI)
Age	1.07 (1.06–1.11)**
Sex	
Male	1 (ref)
Female	1.20 (0.85–1.70)
Year patient was transported	
2001–2006	1 (ref)
2007–2012	0.57 (0.36–0.89)*
2013–2017	0.48 (0.31–0.75)*
Charlson comorbidity score (points)	
0	1 (ref)
1–2	1.65 (1.08–2.53)*
≥ 3	3.36 (1.72–6.54)**
Transport priority	
2–3	1 (ref)
1 (highest priority)	2.92 (1.78–4.79)**
Medical escort required	
No	1 (ref)
Yes	3.22 (1.88–5.51)**
Originating rural health region	
South West	1 (ref)
Kimberley	4.90 (2.23–10.78)**
Pilbara	1.60 (0.78–3.30)
Midwest	1.60 (0.95–2.70)
Wheatbelt	1.58 (0.89–2.82)
Goldfields	1.05 (0.53–2.08)
Great Southern	1.63 (0.93–2.86)

Abbreviation: CI = confidence interval. Note : Significance of *indicates statistical significance of *p* value when *p < 0.05; **p < 0.001.

Some 11 126 transports were admitted to a Perth hospital under acute care with a median LOS of 4 days (25th, 75th percentiles = 3, 7) (Table 3). One percent of these patients (*n* = 122) were also admitted for rehabilitative care during their hospitalisation in Perth. Most (84.5%; *n* = 9398) of these patients received a diagnostic coronary artery procedure. Those transported in 2013–2017 compared to those in 2001–2006 (RR = 1.03; CI = 1.00–1.05) and those with the highest priority transport (RR = 1.04; CI = 1.01–1.07) were more likely to receive a coronary artery procedure (Table 4). Conversely, females (RR = 0.97; CI = 0.95–0.99), those with more comorbidities (RR = 0.81; CI = 0.48–0.83 for Charlson comorbidity scores ≥ 3 compared to 0), from the Pilbara, Midwest and Wheatbelt regions (compared to the South West region) and those who died during their hospitalisation in Perth (RR = 0.56; CI = 0.48–0.66) were less likely to receive this procedure.

**TABLE 3:** Care received during hospitalisation in Perth following transport by RFDSWO for suspected ACS between 2001 and 2017 (*N* = 11 126).

	All rural health regions combined (*N* = 11 126)
Care type at metropolitan hospital: acute care, *n* (%)	11 126 (100)
Length of stay (days) at metropolitan hospital, median (25th, 75th percentiles)	4 (3, 7)
Received rehabilitative care at metropolitan hospital, *n* (%)	122 (1.1)
Coronary artery procedures performed following transfer to metropolitan hospital, *n* (%)	
Coronary angiography^a^	9398 (84.5)
Percutaneous coronary intervention	4591 (41.3)
Coronary artery bypass graft	725 (6.5)

^a^ Assumed received coronary angiography if patient received percutaneous coronary intervention or coronary artery bypass graft.

**TABLE 4:** Independent associations between patient and transport characteristics and receipt of coronary artery procedure during hospitalisation in Perth following transport by RFDSWO for suspected ACS between 2001 and 2017 (*N* = 11 126).

	Risk ratio (95% CI)
Age	1.00 (0.99–1.00)
Sex	
Male	1 (ref)
Female	0.97 (0.95–0.99)*
Year patient was transported	
2001–2006	1 (ref)
2007–2012	1.01 (0.99–1.03)
2013–2017	1.03 (1.00–1.05)*
Charlson comorbidity score (points)	
0	1 (ref)
1–2	0.94 (0.93–0.96)**
≥ 3	0.81 (0.78–0.83)**
Transport priority	
2–3	1 (ref)
1 (highest priority)	1.04 (1.01–1.07)*
Medical escort required	
No	1 (ref)
Yes	0.99 (0.97–1.00)
Originating rural health region	
South West	1 (ref)
Kimberley	1.00 (0.97–1.03)
Pilbara	0.97 (0.94–0.99)*
Midwest	0.93 (0.90–0.95)**
Wheatbelt	0.86 (0.82–0.89)**
Goldfields	0.99 (0.97–1.02)
Great Southern	1.01 (0.99–1.03)
Survival status at discharge from a Perth hospital	
Survived	1 (ref)
Died	0.56 (0.48–0.66)**

Abbreviation: CI = confidence interval. Note : Significance of *indicates statistical significance of *p* value when *p < 0.05; **p < 0.001.

### 3.4 Principal Discharge Diagnosis From Metropolitan Hospital

Of the 11 126 patients who were hospitalised following transport, 8289 (74.5%) were discharged with a principal diagnosis relating to ACS (Table S3). Another 13.9% (*n* = 1551) had a principal discharge diagnosis relating to ‘Other coronary heart disease (CHD)/chest pain’.

### 3.5 Sensitivity Analyses Limited to Transports in the Last 5 Years

Our findings for the last 5 years were consistent with the findings of the entire study period. In addition, we included the variable ‘ACS sub‐type’ in the analysis. Survival was higher in transportations where the patient was diagnosed with suspected UA (Tables S4 and S5). Over 90% of patients transported with a diagnosis of suspected STEMI received a coronary angiography procedure at the metropolitan hospital compared to 72.2% for those with a diagnosis of suspected UA (Table S6). In the multivariable regression model, those with a diagnosis of suspected UA were less likely (RR = 0.82; CI: 0.79–0.85) than those with STEMI to receive a coronary artery procedure during their hospitalisation in Perth (Table S7).

## 4 Discussion

This is the first study describing the counts and outcomes following transfer to Perth by RFDSWO for suspected ACS. Between 2001 and 2017, the number of transports to Perth for suspected ACS increased by an absolute 6.0% per year although the number of ACS in Australia declined between 2001 and 2020 [1]. It is likely that more patients with ACS are transferred to percutaneous coronary intervention‐capable hospitals in Perth, through the implementation of improved ACS management pathways, to facilitate improved patient outcomes [17, 18]. The decline in the number of ACS transfers from the South West after 2013 may be explained by the opening of an eight‐bed coronary care unit in February 2013 in Bunbury (largest population‐centre in the South West) that is capable of performing diagnostic coronary angiography, coronary reperfusion therapy, and more recently percutaneous coronary intervention procedures. Additionally, during the 2013–2014 financial year, St John Ambulance WA established a dedicated Patient Transfer Service by road ambulance (2 h) and increased the number of paramedics in the South West [19], which presented an alternative mode of transport for ACS patients not covered in the RFDSWO data.

Overall, the age and sex distribution of patients is consistent with our earlier rural state‐wide study of patients with ischaemic heart disease (IHD) [2]. However, the prevalence of comorbid conditions in this cohort of RFDSWO patients was lower than our earlier study. For example, the prevalence of congestive heart failure (8.8%) and chronic pulmonary disease (0.4%) was lower than that of the statewide rural patients with IHD (15.4% vs. 11.2%, respectively). This could suggest that patients with more comorbid conditions were less likely to be referred for transfer.

Although a small number of patients died before reaching a metropolitan hospital, most (99%) of the remaining transported cases were hospitalised in Perth for at least 4 days. We found that the vast majority of hospitalised patients received either diagnostic (84%) or interventional (47.8%) cardiac care. Those who were transported under the highest priority were more likely to receive a coronary artery procedure than those under lower priority codes. Furthermore, three‐quarters of patients were diagnosed as ACS and a further 14% with ‘other CHD’ or ‘chest pain’. Together, these indicate a high suspicion of coronary artery pathology among the transported patients and the need to expedite the transfer to offer guideline‐indicated treatment goals. Consistent with the literature, we found that females were less likely to receive a coronary artery procedure than males [15, 20, 21], together with those with higher acuity (based on requirement for medical escort) and more comorbidities. Importantly, we also found that the transfers from the Pilbara, Midwest and Wheatbelt regions were less likely to receive a coronary procedure than their counterparts in the South West. There is some selection bias from the South West region due to its proximity to Perth (< 2 h by road), where less severe patients may be transported by road ambulance rather than by air, resulting in higher numbers of coronary artery procedures observed among RFDSWO‐transported patients from the South West region. Additional factors could include unmeasured comorbidities and the larger Indigenous population in these areas compared to the South West. It was not possible to ascertain Indigenous status in this study's dataset. However, our earlier work among rural patients with IHD showed that Indigenous people were less likely to receive a coronary artery procedure than non‐Indigenous people [2]. Furthermore, the setting and context of the transported case is unknown from the available data. For example, patients could have been transported because electrocardiogram or troponin point‐of‐care testing was not available, or treatment goals were not known at the time in the field but were determined following specialist consult in Perth.

Since 2008, the proportion of transfers with a suspected diagnosis of NSTEMI increased, which is consistent with a previous WA study [11]. This pattern has been observed in several studies, with rising NSTEMI cases attributed to more sensitive troponin testing and updated recommendations for early invasive intervention in higher risk subgroups which therefore necessitate transfer to percutaneous coronary intervention‐capable centres [11, 17, 22]. This recommendation is indirectly supported by a more recent study that found over two‐thirds of rural WA patients transferred for NSTEMI who underwent coronary angiography had severe coronary artery stenosis and over half required revascularisation [23].

Two percent of patients died following their transport to Perth. Compared to those who survived, those who died were older, more likely to have been transferred under highest priority, more likely to require a medical escort, and had more comorbidities. This probably reflects the perceived severity of their condition by the requesting health service. However, it is worth noting that the decision to assign highest priority transfer may not reflect only disease severity but also the capability of the originating hospital in managing the patient. Furthermore, we found that survival to discharge among this cohort of patients with suspected ACS was higher in later years, consistent with earlier reports showing decreased MI case fatality [24, 25]. The lower survival for patients from the Kimberley (which covers remote areas) compared to the South West region could be due to several factors including longer time from onset of symptoms to first presentation, time from presentation to thrombolysis where administered, time from presentation to time of referral to transfer, time from referral transfer and time to actual transfer and the duration of the aeromedical transport. All these potential covariates will require access to more granular data like detailed clinical notes which were not available in this current analysis.

The strengths of this study are its use of person‐linked data that allowed the tracking of care and their outcomes. There are several limitations to this study. Firstly, it only included patients who were transported to Perth and did not include those already confirmed not to have an ACS, those determined unlikely to benefit from transfer, those who declined transportation, and those transferred by road ambulance. Therefore, we have underestimated the true burden of ACS to RFDSWO. Secondly, our dataset does not specify whether the diagnosis instigating the transfer was provided by RFDSWO or the clinician at the referring hospital. Diagnosis could have been more difficult for patients in non‐hospital settings.

## 5 Conclusion

Our findings suggest a high suspicion of coronary artery pathology among transported patients and the need to expedite transfers to offer guideline‐indicated treatment goals. Whilst the groups of people who were less likely to survive or receive a coronary artery procedure are consistent with the literature, the setting and context of decisions are largely based on the RFDS clinician's judgement. Many of these patients have complex medical needs, and clinicians in the field make decisions based on information available to them at the time, sometimes with limited access to diagnostic aids. Distance from definitive care, alternative transport availability and local health service capabilities are also pertinent. Further studies with more granular data are needed to investigate those who did not receive a coronary artery procedure and the small number of patients who died to guide improvements in patient selection and care.

## Author Contributions

**Julian Ming:** formal analysis, writing – original draft, writing – review and editing, visualisation. **Helen Bartholomew:** data curation, project management, study design, methodology, writing – review and editing. **David Preen:** conceptualisation, supervision, resources, study design, methodology, writing – review and editing. **John Fisher:** conceptualisation, supervision, writing – review and editing. **Tom Briffa:** conceptualisation, study design, methodology, writing – review and editing. **Andrew Hooper:** conceptualisation, writing – review and editing. **James M. Rankin:** conceptualisation, writing – review and editing. **Abdul Rahman Ihdayhid:** conceptualisation, writing – review and editing. **Derrick Lopez:** conceptualisation, data curation, supervision, project management, study design, methodology, formal analysis, writing – review and editing, visualisation.

## Ethics Statement

Human Research Ethics Committee approval for the project was obtained from the WA Department of Health (RGS0000003686) and The University of Western Australia (RA/4/20/6520).

## Conflicts of Interest

The authors declare no conflicts of interest.

## Supporting information


**Figure S1:** Patient flow from rural Western Australia to Perth for suspected ACS between 2001 and 2017 (*n* represents number of transport events).
**Figure S2:** Number of RFDSWO transports to Perth for suspected ACS between 2001 and 2017: by rural health region.
**Figure S3:** ACS sub‐types in RFDSWO dataset for transports to Perth between 2001 and 2017: by rural health region.
**Table S1:** Codes for identifying ACS subtypes and coronary artery procedures in the HMDC.
**Table S2:** Characteristics of patients and transports by survival status for RFDSWO transportations between 2001 and 2017 (*N* = 11 226).
**Table S3:** Principal discharge diagnosis from Perth hospitals following RFDSWO transport for suspected ACS (*N* = 11 126)^(a)^.
**Table S4:** Characteristics of patients and transports by survival status for RFDSWO transportations between 2013 and 2017 (*N* = 4690).
**Table S5:** Independent associations between patient/transport characteristics and death during the RFDSWO transportation episode for suspected ACS between 2013 and 2017 (*N* = 4690).
**Table S6:** Care received following transfer to metropolitan hospital for a suspected ACS between 2013 and 2017 and by sub‐type of ACS as classified by RFDSWO (*N* = 4648).
**Table S7:** Independent associations between patient and transport characteristics and receipt of coronary artery procedure following hospitalisation in Perth for suspected ACS between 2013 and 2017 (*N* = 4648).

## Data Availability

The datasets generated and/or analysed during the current study are not publicly available due to the terms of the ethics approval granted by the Western Australian Department of Health Human Research Ethics Committee and data disclosure policies of the Data Providers. The datasets may be available from the corresponding author upon request and subject to approval from the WADOH HREC and relevant custodians.
