# Supplementary material for: Counts, Characteristics and Outcomes of Patients Transported by the Royal Flying Doctor Service to Metropolitan Perth With Suspected Acute Coronary Syndrome: Western Australian Linked Data Study
Source: Aust J Rural Health. 2025 Sep 23;33(5):e70093. doi: 10.1111/ajr.70093 (PMC12455902; doi:10.1111/ajr.70093)
Supplement: Supplementary file 1 — Figure S1: Patient flow from rural Western Australia to Perth for suspected ACS between 2001 and 2017 (n represents number of transport events). Figure S2: Number of RFDSWO transports to Perth for suspected ACS between 2001 and 2017: by rural health region. Figure S3: ACS sub‐types in RFDSWO dataset for transports to Perth between 2001 and 2017: by rural health region. Table S1: Codes for identifying ACS subtypes and coronary artery procedures in the HMDC. Table S2: Characteristics of patients and transports by survival status for RFDSWO transportations between 2001 and 2017 (N = 11 226). Table S3: Principal discharge diagnosis from Perth hospitals following RFDSWO transport for suspected ACS (N = 11 126)(a). Table S4: Characteristics of patients and transports by survival status for RFDSWO transportations between 2013 and 2017 (N = 4690). Table S5: Independent associations between patient/transport characteristics and death during the RFDSWO transportation episode for suspected ACS between 2013 and 2017 (N = 4690). Table S6: Care received following transfer to metropolitan hospital for a suspected ACS between 2013 and 2017 and by sub‐type of ACS as classified by RFDSWO (N = 4648). Table S7: Independent associations between patient and transport characteristics and receipt of coronary artery procedure following hospitalisation in Perth for suspected ACS between 2013 and 2017 (N = 4648). [file AJR-33-0-s001.docx]

**Counts, characteristics and outcomes of patients transported by the Royal Flying Doctor Service to metropolitan Perth with suspected acute coronary syndrome: Western Australian linked data study**

**SUPPLEMENTARY DOCUMENT**

Supplementary Figure 1: Patient flow from rural Western Australia to Perth for suspected ACS between 2001 and 2017 (n represents number of transport events)

ED=Emergency Department; RFDSWO=Royal Flying Doctor Service Western Operations

Supplementary Figure 2: Number of RFDSWO transports to Perth for suspected ACS between 2001 and 2017: by rural health region


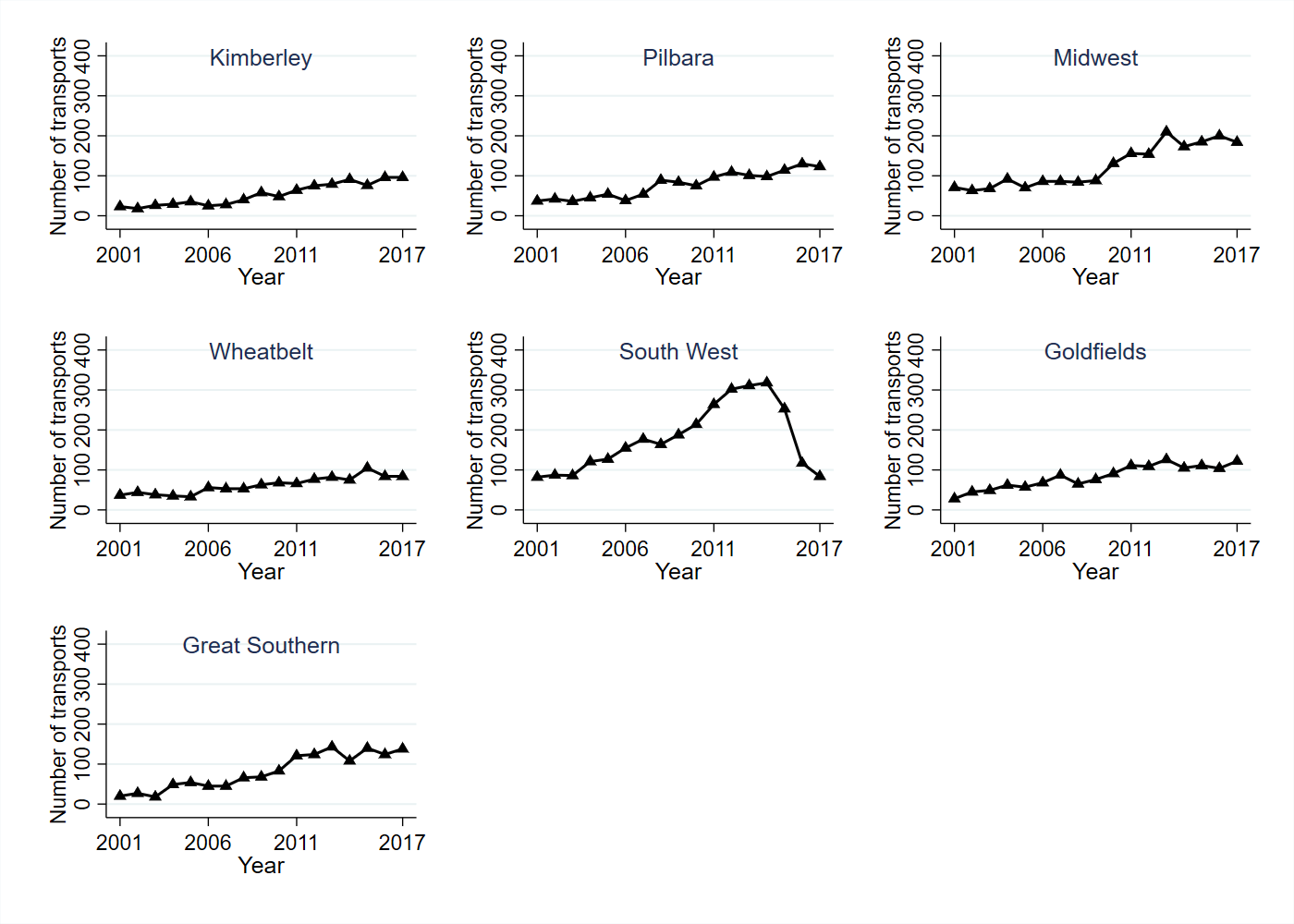


Supplementary Figure 3: ACS sub-types in RFDSWO dataset for transports to Perth between 2001 and 2017: by rural health region


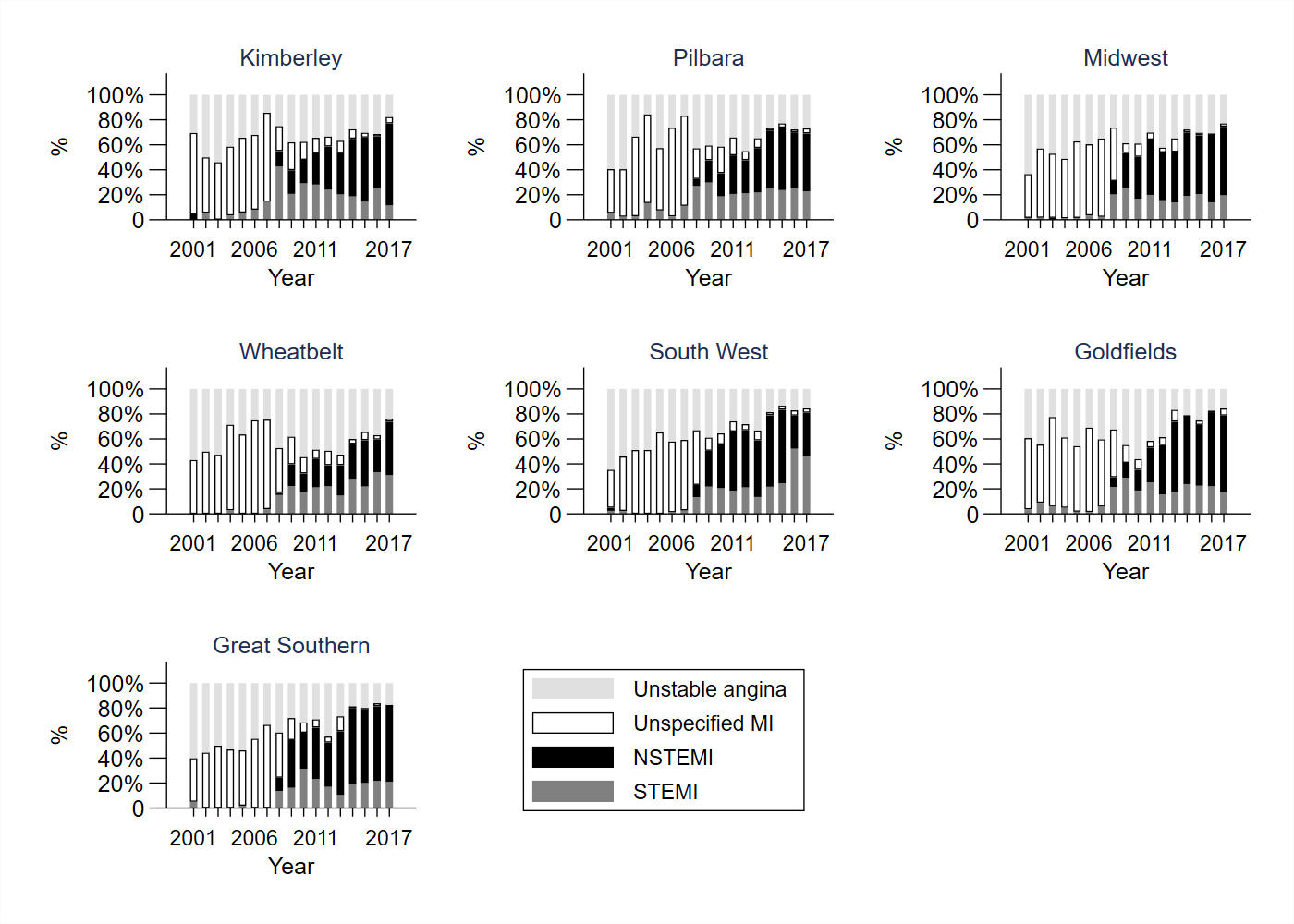


ACS=acute coronary syndrome; MI=myocardial infarction; NSTEMI=non-ST-elevation myocardial infarction; STEMI=ST-elevation myocardial infarction

Supplementary Table 1: Codes for identifying ACS subtypes and coronary artery procedures in the HMDC

| **ACS sub-type** | **ICD 9-CM/ICD 10-AM codes** |
| --- | --- |
|  |  |
| ST-elevation myocardial infarction | ICD 9-CM: 410.0-410.6, 410.8  ICD 10-AM: I21.0-I21.3 |
|  |  |
| Non-ST-elevation myocardial infarction | ICD 9-CM: 410.7  ICD 10-AM: I24 |
|  |  |
| Unspecified myocardial infarction | ICD 9-CM: 410 410.9  ICD 10-AM: I21, I21.9 |
|  |  |
| Unstable angina | ICD 9-CM: 411.1  ICD 10-AM: I20.0 |
|  |  |
| **Coronary artery procedure** | **ACHI code** |
|  |  |
| Coronary angiography | 38215-00, 38218-00, 38218-01, 38218-02, 57360-00 |
|  |  |
| Percutaneous coronary intervention (PCI) | 35304-00, 35305-00, 38300-00, 38303-00, 35310-00, 35310-01, 35310-02, 38306-00, 38306-01, 38306-02, 35335-00, 35341-00, 38309-00, 38315-00, 35338-00, 35338-01, 35344-00, 35344-01, 38312-00, 38312-01, 38318-00, 38318-01, 90218-00, 90218-01, 90218-02, 90218-03, 35304-01, 35305-01, 38300-01, 38303-01, 35310-03, 35310-04, 35310-05, 38306-03, 38306-04, 38306-05 |
|  |  |
| Coronary artery bypass graft (CABG) | 38497-00, 38497-01, 38497-02, 38497-03, 38497-04, 38497-05, 38497-06, 38497-07, 38500-00, 38500-01, 38503-00, 38503-01, 38500-02, 38500-03, 38503-02, 38503-03, 38500-04, 38503-04, 90201-00, 90201-01, 90201-02, 90201-03, 38500-05, 38503-05 |
|  |  |

ACHI= Australian Classification of Health Interventions; ICD 9-CM=International Classification of Diseases, Ninth Revision, Clinical Modification; ICD 10-AM= International Classification of Diseases, Tenth Revision, Australian Modification

Supplementary Table 2: Characteristics of patients and transports by survival status for RFDSWO transportations between 2001 and 2017 (N=11,226)

|  | Survival status of transportation episode | | | |
| --- | --- | --- | --- | --- |
|  | Survived (n=11,020) | Died  (n=206) |  | p-value |
|  |  |  |  |  |
| Age in years, mean (95% CI) ^(a)^ | 60.1 (59.9-60.4) | 70.7 (68.9-72.6) |  | <0.001 |
|  |  |  |  |  |
| Sex: male, n (%) ^(b)^ | 7,811 (70.9) | 134 (65.0) |  | 0.068 |
|  |  |  |  |  |
| Transport priority, n (%) ^(b)^ |  |  |  | <0.001 |
| 2-3 | 10,222 (92.8) | 174 (84.5) |  |  |
| 1 (highest priority) | 798 (7.2) | 32 (15.5) |  |  |
|  |  |  |  |  |
| Medical escort required, n (%) ^(b)^ | 7,474 (67.8) | 175 (85.0) |  | <0.001 |
|  |  |  |  |  |
| Charlson comorbidity score (points), n (%) ^(b)^ |  |  |  | <0.001 |
| 0 | 6,377 (57.9) | 73 (35.4) |  |  |
| 1-2 | 2.974 (27.0) | 62 (30.1) |  |  |
| ≥3 | 1,669 (15.1) | 71 (34.5) |  |  |
|  |  |  |  |  |
| Year patient was transported ^(b)^ |  |  |  | 0.458 |
| 2001-2006 | 2,232 (20.3) | 49 (23.8) |  |  |
| 2007-2012 | 4,180 (37.9) | 75 (36.1) |  |  |
| 2013-2017 | 4,608 (41.8) | 82 (39.8) |  |  |
|  |  |  |  |  |
| Originating rural health region ^(b)^ |  |  |  | <0.001 |
| South West | 3,003 (27.3) | 47 (22.8) |  |  |
| Kimberley | 878 (8.0) | 29 (14.1) |  |  |
| Pilbara | 1312 (11.9) | 14 (6.8) |  |  |
| Midwest | 2,059 (18.7) | 42 (20.4) |  |  |
| Wheatbelt | 1,023 (9.3) | 30 (14.6) |  |  |
| Goldfields | 1,402 (12.7) | 14 (6.8) |  |  |
| Great Southern | 1,343 (12.2) | 30 (14.6) |  |  |
|  |  |  |  |  |

(a) statistical significance based on t-tests; (b) statistical significance based on chi-squared tests; CI=confidence interval

Supplementary Table 3: Principal discharge diagnosis from Perth hospitals following RFDSWO transport for suspected ACS (N=11,126) ^(a)^

| **Principal discharge diagnosis** | **Description** | **n (%)** | |
| --- | --- | --- | --- |
|  |  |  | |
| **ACS-related diagnosis** | |  |  |
| I21.4 | Non-ST-elevation myocardial infarction | 3,986 (35.8) | |
| I21.0-I21.3 | ST-elevation myocardial infarction | 2,900 (26.1) | |
| I21.0 | Unstable angina | 1,301 (11.7) | |
| I21.9 | Unspecified myocardial infarction | 102 (0.9) | |
|  | *Total ACS-related diagnosis* | *8,289 (74.5)* | |
|  |  |  | |
| **Other CHD/chest pain** | |  | |
| R07.1-R07.4 | Chest pain | 971 (8.7) | |
| I20.1, I20.8, I20.9 | Stable angina | 315 (2.8) | |
| I25.x | Chronic ischaemic heart disease | 216 (1.9) | |
| I22.x | Subsequent myocardial infarction | 34 (0.3) | |
| I24.x | Other acute ischaemic heart diseases | 9 (0.1) | |
| I23.x | Complications following acute myocardial infarction | 6 (0.1) | |
|  | *Total other CHD/chest pain* | *1,551 (13.9)* | |
|  |  |  | |
| **Other CVD** |  |  | |
| I42.x | Cardiomyopathy | 127 (1.1) | |
| I48.x | Atrial fibrillation/flutter | 118 (1.1) | |
| I50.x | Heart failure | 117 (1.1) | |
| I31.x | Other diseases of the pericardium | 80 (0.7) | |
| I47.x | Paroxysmal tachycardia | 48 (0.4) | |
| I30.x | Acute pericarditis | 44 (0.4) | |
| I26.x | Pulmonary embolism | 41 (0.4) | |
| I35.x | Non-rheumatic aortic valve disorders | 39 (0.4) | |
| J51.x | Complications/ill-defined descriptions of heart disease | 22 (0.2) | |
| I49.x | Other cardiac arrhythmias | 20 (0.2) | |
| I40.x | Acute myocarditis | 17 (0.2) | |
| I44.x | Atrioventricular/left bundle-branch block | 14 (0.1) | |
| I97.x | Intraoperative/postprocedural disorders of circulatory system, not elsewhere classified | 14 (0.1) | |
| I63.x | Cerebral infarction | 12 (0.1) | |
| I33.x | Acute/subacute endocarditis | 10 (0.1) | |
| I71.x | Aortic aneurysm/dissection | 10 (0.1) | |
| I08.x | Multiple valve diseases | 6 (0.1) | |
| I95.x | Hypotension | 6 (0.1) | |
| I45.x | Other conduction disorders | 5 (<0.1) | |
| I61.x | Intracerebral haemorrhage | 5 (<0.1) | |
|  | *Total Other CVD* | *786 (7.1)* | |
|  |  |  | |
| **Non-CVD** |  |  | |
| J18.x | Pneumonia, organism unspecified | 32 (0.3) | |
| R55 | Syncope and collapse | 30 (0.3) | |
| J44.x | Other chronic obstructive pulmonary disease | 25 (0.2) | |
| K80.x | Cholelithiasis | 23 (0.2) | |
| K21.X | Gastro-oesophageal reflux disease | 22 (0.2) | |
| K29.x | Gastritis/duodenitis | 20 (0.2) | |
| R00.x | Abnormalities of heart beat | 20 (0.2) | |
| A41.x | Other/unspecified sepsis | 13 (0.1) | |
| E11.x | Type 2 diabetes mellitus | 13 (0.1) | |
| J22 | Unspecified acute lower respiratory infection | 13 (0.1) | |
| E87.x | Other disorders of fluid, electrolyte and acid-base balance | 10 (0.1) | |
| K85.x | Acute pancreatitis | 9 (0.1) | |
| R06.x | Abnormalities of breathing | 9 (0.1) | |
| M94.x | Other disorders of cartilage | 8 (0.1) | |
| T82.x | Complications of cardiac and vascular prosthetic devices, implants and grafts | 8 (0.1) | |
| K22.x | Other diseases of oesophagus | 7 (0.1) | |
| N18.x | Chronic kidney disease | 7 (0.1) | |
| K25.x | Gastric ulcer | 6 (0.1) | |
| M79.x | Other soft tissue disorders, not elsewhere classified | 6 (0.1) | |
| R10.x | Abdominal/pelvic pain | 6 (0.1) | |
| E05.x | Thyrotoxicosis (hyperthyroidism) | 5 (<0.01) | |
| G45.x | Transient cerebral ischaemic attacks and related syndromes | 5 (<0.01) | |
| R74.x | Abnormal serum enzyme levels | 5 (<0.01) | |
|  | *Total non-CVD* | *500 (4.5)* | |
|  |  |  | |

ACS=acute coronary syndrome; CHD=coronary heart disease; CVD=cardiovascular disease; (a) only diagnosis codes with counts ≥5 are presented

Supplementary Table 4: Characteristics of patients and transports by survival status for RFDSWO transportations between 2013-2017 (N=4,690)

|  | Survival status of transportation episode | | | |
| --- | --- | --- | --- | --- |
|  | Survived (n=4,608) | Died  (n=82) |  | p-value |
|  |  |  |  |  |
| Age in years, mean (95% CI) ^(a)^ | 61.0 (60.6-61.4) | 69.7 (66.7-72.7) |  | <0.001 |
|  |  |  |  |  |
| Sex: male, n (%) ^(b)^ | 3,195 (69.3) | 50 (61.0) |  | 0.104 |
|  |  |  |  |  |
| Transport priority, n (%) ^(b)^ |  |  |  | 0.007 |
| 2-3 | 4,250 (92.2) | 69 (84.1) |  |  |
| 1 (highest priority) | 358 (7.8) | 13 (15.9) |  |  |
|  |  |  |  |  |
| Medical escort required, n (%) ^(b)^ | 3,155 (68.5) | 72 (87.8) |  | <0.001 |
|  |  |  |  |  |
| Charlson comorbidity score (points), n (%) ^(b)^ |  |  |  | <0.001 |
| 0 | 2,499 (54.2) | 25 (30.5) |  |  |
| 1-2 | 1.297 (28.1) | 24 (29.3) |  |  |
| ≥3 | 812 (17.6) | 33 (40.2) |  |  |
|  |  |  |  |  |
| ACS sub-type in RFDSWO dataset ^(b)^ |  |  |  | <0.001 |
| STEMI | 972 (21.1) | 25 (30.5) |  |  |
| NSTEMI | 2,262 (49.1) | 40 (48.8) |  |  |
| Unspecified MI | 193 (4.2) | 10 (12.2) |  |  |
| Unstable angina | 1,181 (25.6) | 7(8.5) |  |  |
|  |  |  |  |  |
| Originating rural health region ^(b)^ |  |  |  | 0.079 |
| South West | 1,069 (23.2) | 14 (17.1) |  |  |
| Kimberley | 423 (9.2) | 15 (18.3) |  |  |
| Pilbara | 558 (12.1) | 8 (9.8) |  |  |
| Midwest | 938 (20.4) | 14 (17.1) |  |  |
| Wheatbelt | 419 (9.1) | 11 (13.4) |  |  |
| Goldfields | 560 (12.2) | 8 (9.8) |  |  |
| Great Southern | 641 (13.9) | 12 (14.6) |  |  |
|  |  |  |  |  |

ACS=acute coronary syndrome; CI=confidence interval; MI=myocardial infarction; NSTEMI=non-ST-elevation myocardial infarction; RFDSWO=Royal Flying Doctor Service Western Operations; STEMI=ST-elevation myocardial infarction; (a) statistical significance based on t-tests; (b) statistical significance based on chi-squared tests

Supplementary Table 5: Independent associations between patient/transport characteristics and death during the RFDSWO transportation episode for suspected ACS between 2013 and 2017 (N=4,690)

|  | **Odds ratio (95% CI)** |
| --- | --- |
|  |  |
| Age | **1.07 (1.06-1.10)** |
| Sex |  |
| Male | 1 (ref) |
| Female | 1.37 (0.75-2.50) |
| Charlson comorbidity score (points) |  |
| 0 | 1 (ref) |
| 1-2 | 1.99 (0.97-4.06) |
| ≥3 | **5.00 (2.01-12.44)** |
| Transport priority |  |
| 2-3 | 1 (ref) |
| 1 (highest priority) | 2.22 (0.93-5.30) |
| Medical escort required |  |
| No | 1 (ref) |
| Yes | **4.00 (1.72-9.28)** |
| Originating rural health region |  |
| South West | 1 (ref) |
| Kimberley | **7.07 (2.09-23.89)** |
| Pilbara | 2.54 (0.84-7.69) |
| Midwest | 1.61 (0.64-4.00) |
| Wheatbelt | 2.45 (0.87-6.87) |
| Goldfields | 2.05 (0.70-6.00) |
| Great Southern | 1.83 (0.72-4.61) |
| ACS sub-type in RFDSWO dataset |  |
| STEMI | 1 (ref) |
| NSTEMI | 0.90 (0.43-1.86) |
| Unspecified MI | 2.77 (0.95-8.03) |
| Unstable angina | **0.22 (0.08-0.61)** |
|  |  |

ACS=acute coronary syndrome; CI=confidence interval; MI=myocardial infarction; NSTEMI=non-ST-elevation myocardial infarction; RFDSWO=Royal Flying Doctor Service Western Operations; STEMI=ST-elevation myocardial infarction

Supplementary Table 6: Care received following transfer to metropolitan hospital for a suspected ACS between 2013 and 2017 and by sub-type of ACS as classified by RFDSWO (N=4,648)

|  | All ACS sub-types | STEMI | NSTEMI | Unspecified MI | Unstable angina |
| --- | --- | --- | --- | --- | --- |
|  | n=4,648 | n=993 | N=2,286 | n=198 | n=1,171 |
|  |  |  |  |  |  |
| Care type at metropolitan hospital: acute care, *n (%)* | 4,648 (100) | 993 (100) | 2,286 (100) | 198 (100) | 1,171 (100) |
| Length of stay (days) at metropolitan hospital, *median (25^th^, 75^th^ percentiles)* | 4 (3, 6) | 4 (3, 6) | 4 (3, 7) | 4 (3, 7) | 4 (3, 6) |
| Received rehabilitative care at metropolitan hospital, *n (%)* | 52 (1.1) | 12 (1.2) | 25 (1.1) | <2% ^(b)^ | <2% ^(b)^ |
|  |  |  |  |  |  |
| Coronary artery procedures performed following transfer to metropolitan hospital*, n (%)* |  |  |  |  |  |
| Coronary angiography ^(a)^ | 3,836 (82.5) | 898 (90.4) | 1,927 (84.3) | 166 (83.9) | 845 (72.2) |
| Percutaneous coronary intervention | 1,915 (41.2) | 649 (65.4) | 884 (38.7) | 102 (51.5) | 280 (23.9) |
| Coronary artery bypass graft | 301 (6.5) | 50 (5.0) | 184 (8.0) | 6 (3.0) | 61 (5.2) |
|  |  |  |  |  |  |

ACS=acute coronary syndrome; MI=myocardial infarction; NSTEMI=non-ST-elevation myocardial infarction; STEMI=ST-elevation myocardial infarction; (a) assumed received coronary angiography if patient received percutaneous or coronary artery bypass graft; (b) masked due to small cell counts (<15)

Supplementary Table 7: Independent associations between patient and transport characteristics and receipt of coronary artery procedure following hospitalisation in Perth for suspected ACS between 2013 and 2017 (N=4,648)

|  | **Risk ratio (95% CI)** |
| --- | --- |
|  |  |
| Age | 1.00 (0.99-1.00) |
| Sex |  |
| Male | 1 (ref) |
| Female | **0.96 (0.93-0.98)** |
| Charlson comorbidity score (points) |  |
| 0 | 1 (ref) |
| 1-2 | **0.97 (0.94-0.99)** |
| ≥3 | **0.84 (0.80-0.98)** |
| Transport priority |  |
| 2-3 | 1 (ref) |
| 1 (highest priority) | **1.03 (0.99-1.06)** |
| Medical escort required |  |
| No | 1 (ref) |
| Yes | **0.97 (0.94-0.99)** |
| Originating rural health region |  |
| South West | 1 (ref) |
| Kimberley | **1.05 (1.00-1.09)** |
| Pilbara | 1.00 (0.95-1.04) |
| Midwest | **0.96 (0.93-0.99)** |
| Wheatbelt | **0.94 (0.89-0.99)** |
| Goldfields | 1.00 (0.96-1.04) |
| Great Southern | 1.04 (0.99-1.07) |
| ACS sub-type in RFDSWO dataset |  |
| STEMI | 1 (ref) |
| NSTEMI | **0.94 (0.91-0.97)** |
| Unspecified MI | **0.93 (0.87-0.98)** |
| Unstable angina | **0.82 (0.79-0.85)** |
| Survival status to discharge from hospital |  |
| Survived | 1 (ref) |
| Died | **0.55 (0.43-0.70)** |
|  |  |

ACS=acute coronary syndrome; CI=confidence interval; MI=myocardial infarction; NSTEMI=non-ST-elevation myocardial infarction; RFDSWO=Royal Flying Doctor Service Western Operations; STEMI=ST-elevation myocardial infarction
